# Supplementary material for: Heart Ferroportin Protein Content Is Regulated by Heart Iron Concentration and Systemic Hepcidin Expression
Source: Int J Mol Sci. 2022 May 24;23(11):5899. doi: 10.3390/ijms23115899 (PMC9180074; doi:10.3390/ijms23115899)
Supplement: Supplementary file 1 [file ijms-23-05899-s001.zip › Figure S1.pdf]

Figure S1: Comparison of heart and liver *Hamp* mRNA content.

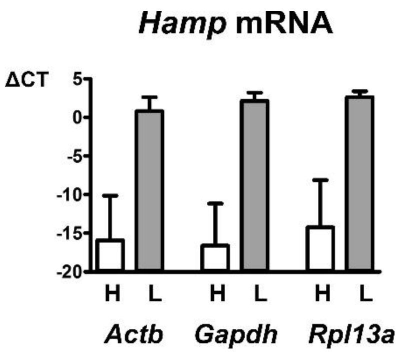

Mouse heart and liver *Hamp* mRNA content was determined by real-time PCR using three reference genes; primer sequences are given in Table S1. Primers used for Hamp mRNA content determination are specific for *Hamp*, they do not amplify murine *Hamp2*. n=3.
