# Supplementary material for: Heart Ferroportin Protein Content Is Regulated by Heart Iron Concentration and Systemic Hepcidin Expression
Source: Int J Mol Sci. 2022 May 24;23(11):5899. doi: 10.3390/ijms23115899 (PMC9180074; doi:10.3390/ijms23115899)
Supplement: Supplementary file 1 [file ijms-23-05899-s001.zip › Figure S2.pdf]

Figure S2: Comparison of heart, liver and spleen *Fpn* mRNA content.

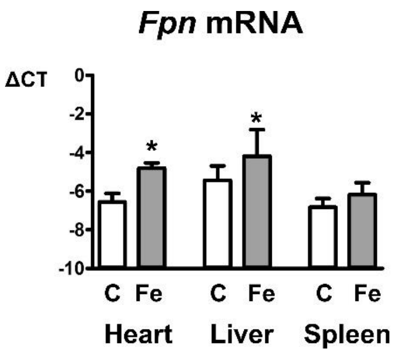

Mouse heart, liver and spleen *Fpn* mRNA content was determined by real-time PCR in control animals and in animals treated injected with iron carboxymaltose at 300 mg Fe/kg. Asterisk denote statistical significance, n=3.
