# Supplementary material for: Heart Ferroportin Protein Content Is Regulated by Heart Iron Concentration and Systemic Hepcidin Expression
Source: Int J Mol Sci. 2022 May 24;23(11):5899. doi: 10.3390/ijms23115899 (PMC9180074; doi:10.3390/ijms23115899)
Supplement: Supplementary file 1 [file ijms-23-05899-s001.zip › Table S1.pdf]

List of primers used for PCR

Mouse:

Actb Forward	GCTACAGCTTCACCACCACA
Actb Reverse	GGTCTTTACGGATGTCAACG
Hamp Forward	CTGAGCAGCACCACCTATCTC
Hamp Reverse	TGGCTCTAGGCTATGTTTTGC
Fpn Forward	ATCGGTCTTTGGTCCTTTGAT
Fpn Reverse	ATTGCCACAAAGGAGACTGAA

Rat:

Actb Forward	TGTCACCAACTGGGACGATA
Actb Reverse	AACACAGCCTGGATGGCTAC
Hamp Forward	GAAGGCAAGATGGCACTAAGCA
Hamp Reverse	TCTCGTCTGTTGCCGGAGATAG
Fpn Forward	ATCGGTCTTTGGTCCTTTGAT
Fpn Reverse	ATTGCCACAAAGGAGACTGAA
